# Efficient cytoplasmic cell quantification using a semi-automated FIJI-based tool

**DOI:** 10.1038/s41598-025-12144-x

**Published:** 2025-07-28

**Authors:** Lucas Unger, Ulrik Larsen, Shayla Sharmine, Md Kaykobad Hossain, Thomas Aga Legøy, Marc Vaudel, Luiza Ghila, Simona Chera

**Affiliations:** 1 Mohn Research Center for Diabetes Precision Medicine, Department of Clinical Science, Faculty of Medicine, University of Bergen, Glasblokkene 1, Haukelandsbakken 15, 5009 Bergen, Norway; 2 Department of Clinical Science, Faculty of Medicine, University of Bergen, Postboks 7804, 5020 Bergen, Norway; 3 Computational Biology Unit, Department of Informatics, University of Bergen, Bergen, Norway

**Keywords:** Cellular imaging, Image processing, Data processing

## Abstract

Quantification of subcellular structures such as nuclei and cytoplasmic proteins using staining methods based on fluorescent dyes or fluorescently tagged antibodies are widely used in scientific research. Accurate high-throughput quantitation of these assays can be time consuming and challenging. Here, we present our FIJI based Semi-Automated counting Macro termed SAM, and we validate its accuracy against manual counting and other automated counting methods. By introducing this automated quantification tool, we aim to contribute to the ongoing efforts to enhance the reliability, efficiency, and standardization of immunostaining analysis in the field of diabetes research and beyond.

**Supplementary Information:**

The online version contains supplementary material available at 10.1038/s41598-025-12144-x.

## Introduction

Immunohistochemistry (IHC) and immunofluorescence (IF) staining techniques are essential tools in biomedical research and clinical diagnostics, enabling the identification and quantification of specific proteins while preserving their spatial context within tissues. Traditionally, the quantification of these staining heavily relies on manual assessment by trained observers. However, this approach is time-consuming, subjective, prone to human error, and susceptible to bias, leading to challenges in reproducibility and standardization^1,2^.

In recent years, there has been a growing recognition for automated quantification methods in the analysis of immunostainings. Automatic quantification can significantly increase throughput and reduce bias in the analysis^3,4^. As these automated systems continue to evolve, they hold the potential to revolutionize quantification and evaluation of immunostained samples by providing more standardized, quantitative, and time-efficient quantitative analyses of complex tissue samples.

While automated cell counting systems have made significant advances in recent years^5,6^, manual counting remains the gold standard in most research fields^7–10^. Automated methods excel in large-scale studies^11^ and when absolute objectivity is crucial^12^. However, these methods may struggle with inadequately processed specimens or complex staining patterns. The decision to use manual or automated counting methods is influenced by the specific needs of the study, sample complexity, staining quality and desired level of accuracy.

Considering these advancements and the persistent need for reliable, efficient, and unbiased quantification methods, we have developed a user-friendly semi-Automated quantitative Macro (SAM) using Fiji (ImageJ) to automatically quantify cytoplasmic expressing cells, with a specific focus on quantifying dense cellular environments, such as the insulin-expressing β-cells in the pancreatic islet. This tool aims to address the limitations of manual quantification by providing a standardized, time-efficient, and objective method for analyzing immunostaining, while allowing the fine and easy tuning of many parameters, which might be inaccessible in pre-tailored pipelines. Moreover, we also designed an intuitive graphical user interface (GUI) to make SAM accessible for newcomers, lowering the barrier for those unfamiliar with automated quantification techniques. This design aims to encourage broader adoption of automated analysis methods in the field.

## Results

### Description of SAM pipeline and graphical user interface

Our technique builds upon the precision of StarDist^13^ for nuclei segmentation and extends its capabilities by analyzing the surrounding cellular area. This is achieved by expanding each detected nucleus by 1 µm in all directions and measuring the staining signal in the resulting interspace. Users can then input a threshold for Fluorescence intensity to determine what constitutes a positive signal. This approach not only identifies cells but also enables the classification of expression levels based on user-defined thresholds.

**Fig. 1:**
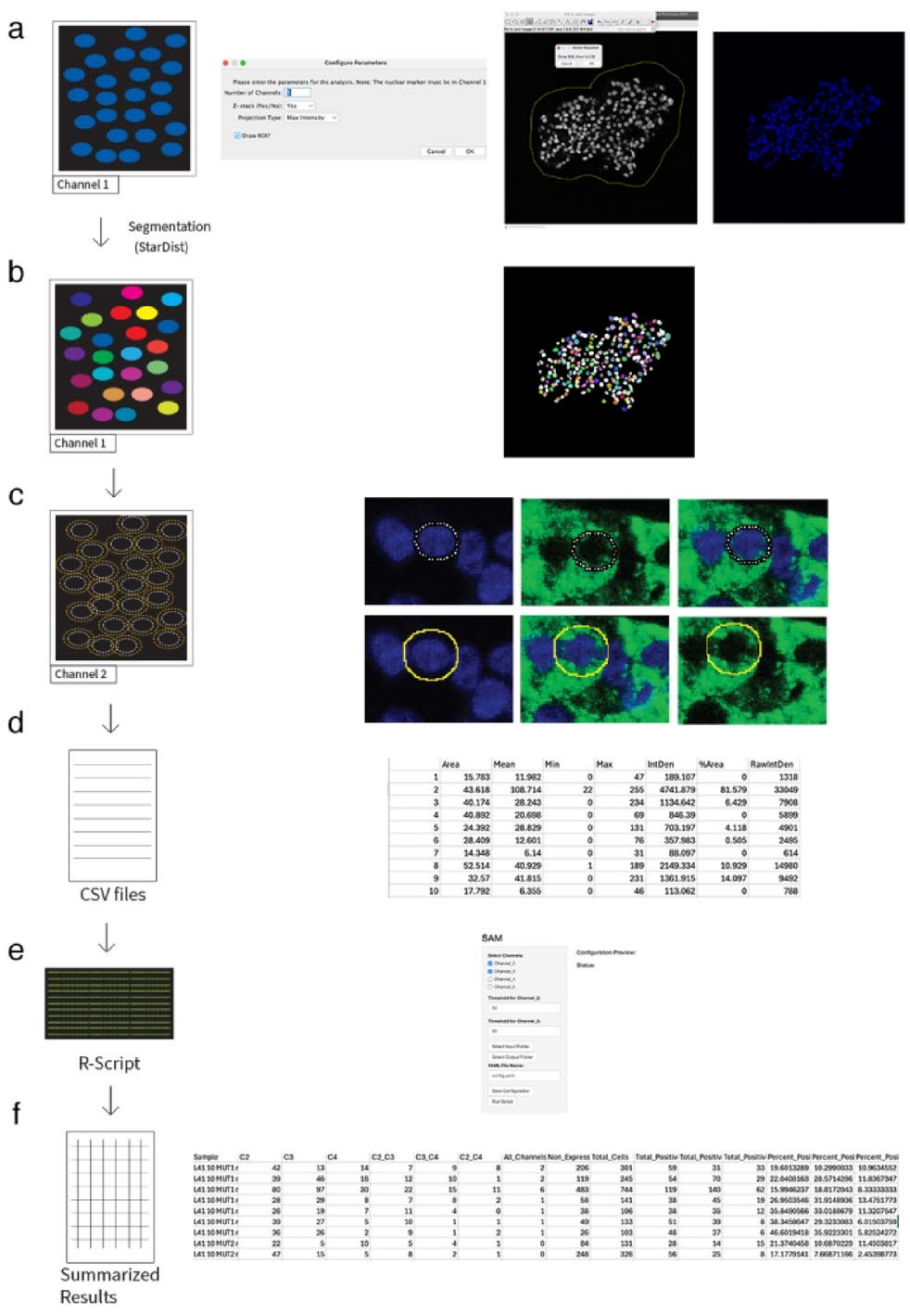
(**a**) Nuclear segmentation in using StarDist. (**b**) Unique labeling of segmented nuclei (**c**) ROI and intensity measurement for DAPI-based ROI and 1 µM enlarged ROI. (**d**) Measurements of quantified data as CSV files. (**e**) Data analysis using an integrated R-script with user-defined parameters. (**f**) Summary of results after processing with SAM R-script.

To illustrate the workflow of our automated quantification method, we provide a conceptual overview of the Macro pipeline (Fig. 1a–f). In a stepwise manner, the user is first prompted to define a single region of interest (ROI) around the objects of interest (Fig. 1a), which will lead to their multicolored masking (Fig. 1b). Subsequently, individual ROIs are created for each nucleus identified within the mask. Each ROI is measured, expanded by 1 µm in all directions, and measured again (Fig. 1c). The intensity values for all measured channels within both the original and expanded ROIs are then saved as a CSV file for each sample (Fig. 1d). Following this, the user executes an R script on a directory containing all CSV files generated by the Fiji macro (Fig. 1e). After specifying the relevant channels and defining thresholds for each, the script outputs summarized results for each sample as a CSV file. These results include the count of all cells positive for the selected channels, including both single positive and multi-positive cells, and the percentage of positive cells relative to the total cell count. (Fig. 1f).

### Rapid and consistent identification of close proximity objects in randomly distributed cell populations

**Fig. 2:**
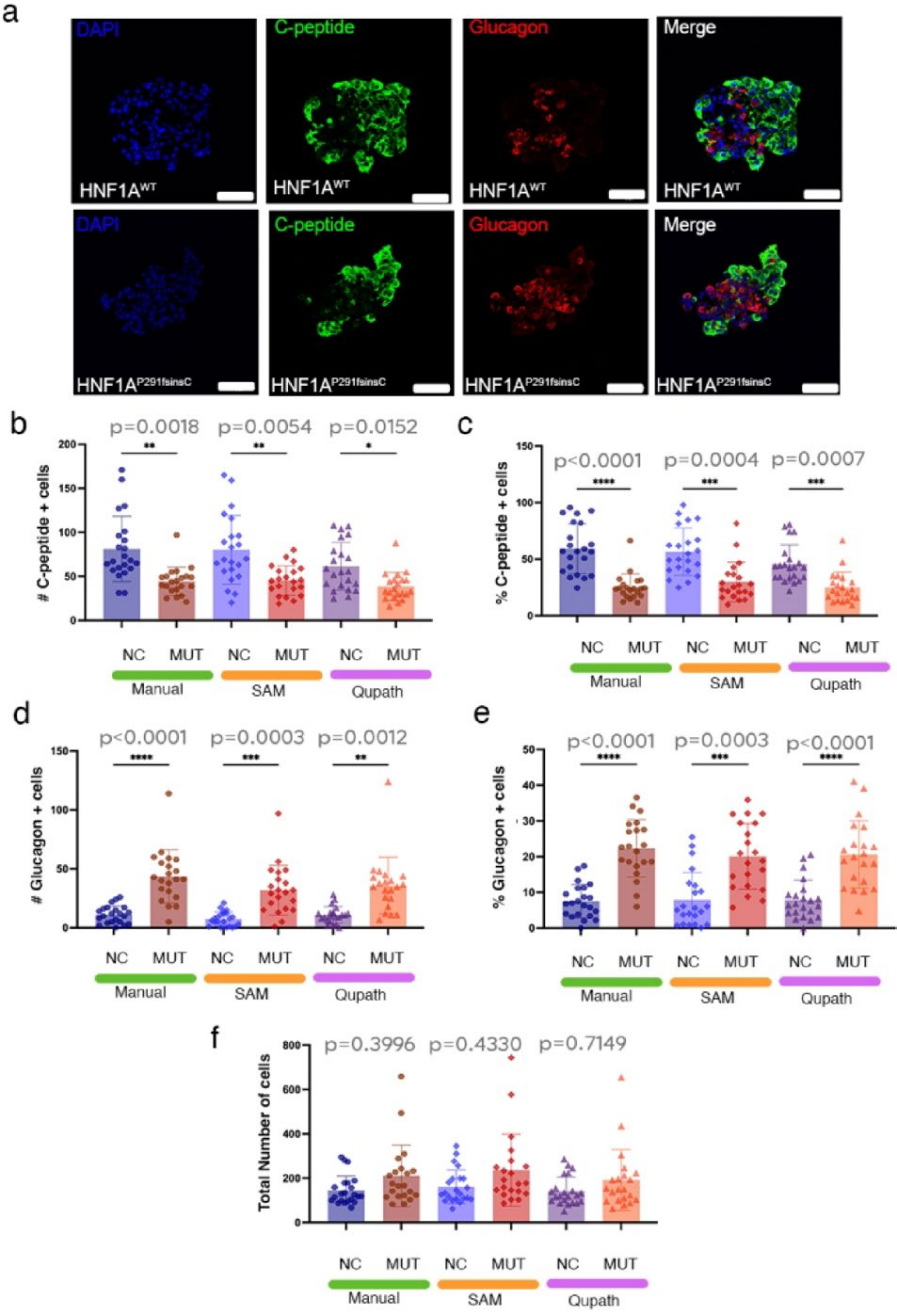
(**a**) Representative immunofluorescence images of pancreatic islets from HNF1A^WT^ (NC) and HNF1A^P291fsInsC^ (MUT) samples stained for DAPI (blue), C-peptide (green, insulin-producing β-cells), and Glucagon (red, glucagon-producing α-cells). Merged images highlight differences in the distribution of endocrine cell populations between genotypes. Scale bars: 50 µm. (**b**–**e**) Quantification of C-peptide + and Glucagon + cells in NC and MUT islets using three methods: manual counting), SAM macro-based analysis, and QuPath-based image processing. (**f**) Total number of cells per islet across NC and MUT samples. (n = 22 sc-islet sections for NC and n = 21 sc-islet sections for MUT).

To test SAM’s reliability in identifying and quantifying densely distributed cell populations, we employed stem cells islets (SC-islets—organoid-like compact structures generated via guided differentiation of human induced pluripotent stem cells (hiPSC) towards pancreatic endocrine cell fate^14–18^). These mainly consist of several distinct neuroendocrine cell types, such as glucagon-producing α-cells and insulin-producing β-cells. To further challenge the robustness of the quantification, we employed SC-islets derived from either normal (NC) or CRISPR-edited hiPSC bearing the HNF1A^P291fsInsC^ mutation (MUT, Fig. 2a). HNF1A is an important regulator of both islet cell fate and function, its dysfunction leading to HNF1A-MODY (formerly known as MODY3—Maturity Onset Diabetes of the Young)^19,20^, an autosomal dominant diabetes disorder characterized by decreased β-cell mass and increase α-cell mass amongst others^21–24^.

In the above contexts, the comparison between manual counting, SAM and the open-source image processing software QuPath^25^, indicated very similar results. All three methods successfully identified a significant decrease in C-peptide + (β-cell marker) cell numbers (Fig. 2b, *p* = 0.0018^[manual]^, 0.0054^**[SAM**]^, 0.0152^[QuPath]^) and percentage (Fig. 2c, *p* < 0.0001^[manual]^, 0.0004^**[SAM**]^, 0.0007^[QuPath]^). Moreover, all successfully identified the increase of Glucagon + (α-cell marker) cells numbers (Fig. 2d, *p* < 0.0001^[manual]^, 0.0003^**[SAM**]^, 0.0012^[QuPath]^) and percentage (Fig. 2e, *p* < 0.0001^[manual]^, 0.0003^**[SAM**]^, 0.0001^[QuPath]^), while the total number of SC-islets cells was found unchanged (Fig. 2f, *p* < 0.3996^[manual]^, 0.4330^**[SAM**]^, 0.7149^[QuPath]^).

Overall, these results suggest that SAM can correctly identify close proximity target objects, closely mirroring the manual method’s outcomes. Importantly, the similar total number of cells per islet between normal and mutant SC-islets confirmed that the observed trends in C-peptide + and Glucagon + cell quantification are consistent across methodologies and are not caused by variations in total cell counts.

### Automated identification of high and low expressing cells using SAM

**Fig. 3:**
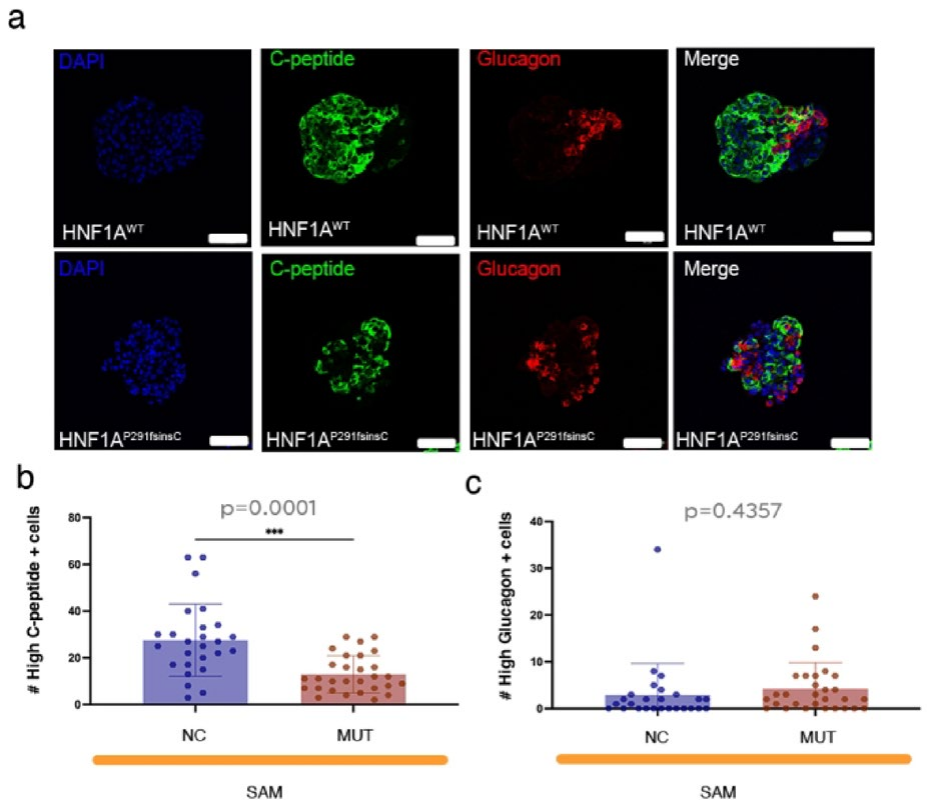
(**a**) Representative immunofluorescence images of pancreatic islets with varying expression levels from HNF1A^WT^ (NC) and HNF1A^P291fsInsC^ (MUT) samples stained for DAPI (blue), C-peptide (green, insulin-producing β-cells), and Glucagon (red, glucagon-producing α-cells). Scale bars: 50 µm. (**b**) Quantification of High expressing C-peptide + cells in NC and MUT islets using SAM macro-based analysis. (**c**) Quantification of High expressing Glucagon + cells in NC and MUT islets using SAM macro-based analysis. (n = 22 sc-islet sections for NC and n = 21 sc-islet sections for MUT).

Manually identifying populations with varying expression levels is difficult, especially in dense populations with heterogeneous intensity profiles. To assess SAM’s ability to perform this task, we define specific thresholds for high-expressing cells and analyze the same batch of images as in the previous assessments (Fig. 3a, see also Fig. 2).

SAM identifies a significant reduction in the number of C-peptide + cells in HNF1A-MODY SC-islets (Fig. 3b, *p* = 0.001), consistent with the overall decrease in peptide-expressing cells under these conditions (see Fig. 2b). However, no significant difference is found in the Glucagon + cell population (Fig. 3c, *p* = 0.4357), deviating from the trend observed in the total Glucagon + population (Fig. 2d).

These findings demonstrate SAM’s ability to distinguish subpopulations with different expression intensity profiles, a task that remains challenging for human observers. Interestingly, the trend in high-expressing Glucagon + cells does not align with that of the overall Glucagon + population, offering further insights into Glucagon + cell dynamics in a biological context.

### SAM quantification of well characterized structures successfully parallels previous reports

**Fig. 4:**
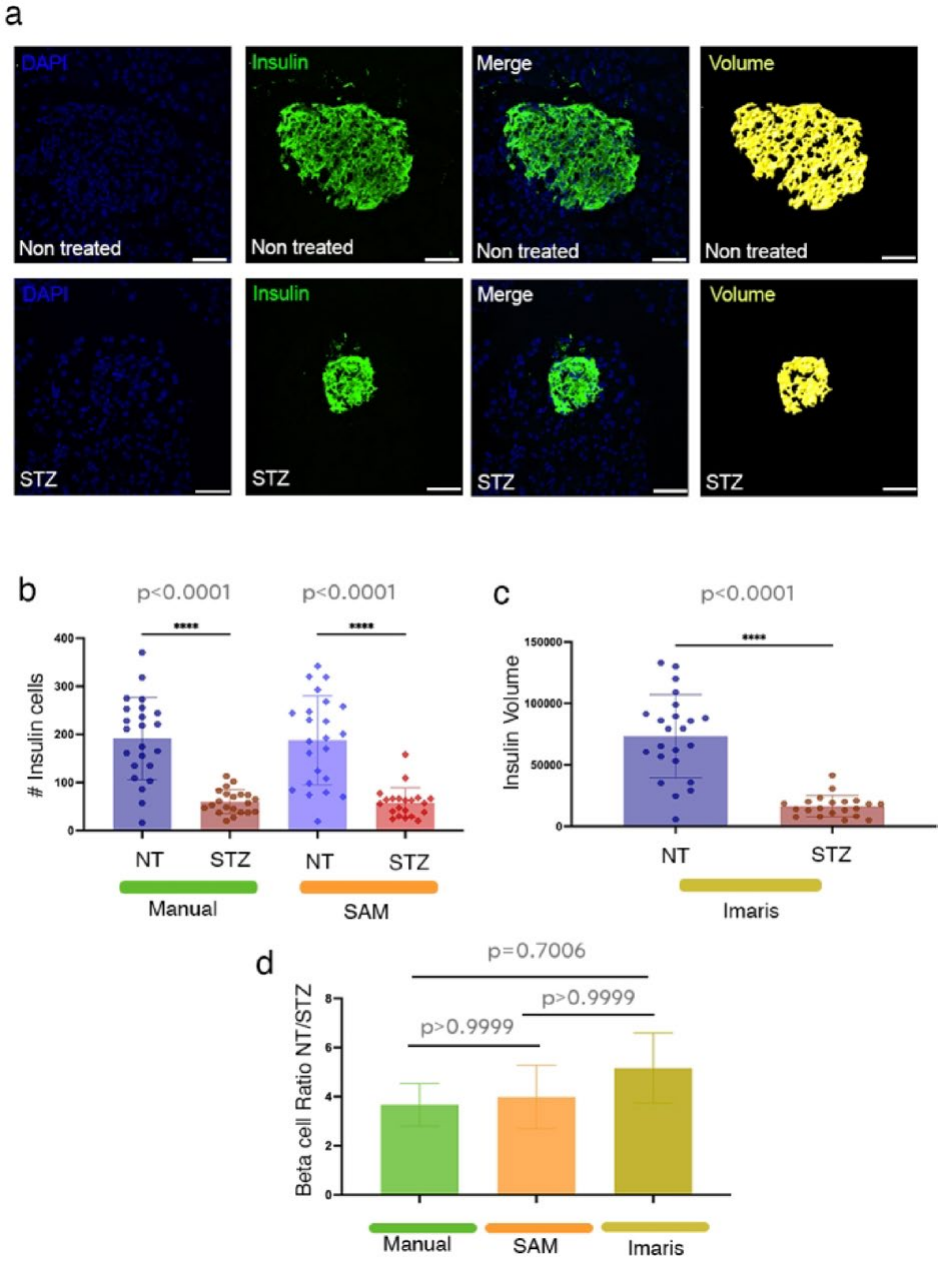
Pancreatic mouse tissue sections from non-treated (NT) and streptozotocin (STZ)-treated mice to evaluate the detection of insulin-producing β-cells. (**a**) Representative images with insulin + cells (green), corresponding Volume from Imaris (yellow) and DAPI(blue) within pancreatic islets. Scale bars: 50 µm. (**b**) Quantification of Insulin + cells in NT and STZ islets using Manual and SAM macro-based analysis (n = 22 islet sections for NT and n = 21 islet sections for STZ). (**c**) Quantification of Insulin Volume in NT and STZ islets using Imars. (n = 23 islet sections for NT and n = 21 islet sections for STZ). (**d**) Comparison of Beta cell ratio from NT to STZ for the three different quantification methods: Manual, SAM and Imaris. Brown-Forsythe and Welch ANOVA tests were used (n = 3 mice per condition).

To further test the quantification robustness, we focused on murine pancreatic islets, which benefit from a detailed previous architecture characterization^26–30^. In this well-described context, we used IF on pancreatic tissue sections from non-treated (NT) and streptozotocin (STZ)-treated mice to evaluate the detection of insulin-producing β-cells (Fig. 4a). STZ is a glucosamine-nitrosourea that targets and destroys β-cells in a dose-dependent manner, thus critically decreasing the β-cell mass^31–34^, the volume rendering further highlighting the structural organization of insulin-producing cells (Fig. 4a, lower row, yellow mask).

To assess β-cell composition, insulin + cell numbers and total insulin volume were quantified using manual counting (gold standard), SAM and Imaris (commercial high-end image-analysis software) 3D volume quantification. All three approaches reliably detected and quantified β-cell populations in NT and STZ-treated samples. SAM closely mirrored manual quantification, yet with increased processing efficiency (Fig. 4b, *p* < 0.0001^[manual]^, 0.0001^**[SAM**]^), while the Imaris-based analysis offered a complementary volumetric assessment (Fig. 4c, *p* < 0.0001^[Imaris]^), further confirming the observed trends.

As expected, based on the previous literature, the statistical analysis demonstrated a significant reduction in insulin + cell number and volume in STZ-treated samples compared to NT controls (*p* < 0.0001 for all methods), with consistent results across the three quantification strategies (Fig. 4b, c). The ratio of β-cells in NT versus STZ conditions was comparable in all methods, reinforcing the robustness of SAM’s approach (Fig. 4d).

These findings further validate the reliability of the macro-based analysis for β-cell quantification in pancreatic tissue sections, demonstrating its utility alongside manual counting and volumetric assessment tools.

### Reliable quantification in structures with unclear regional borders

**Fig. 5:**
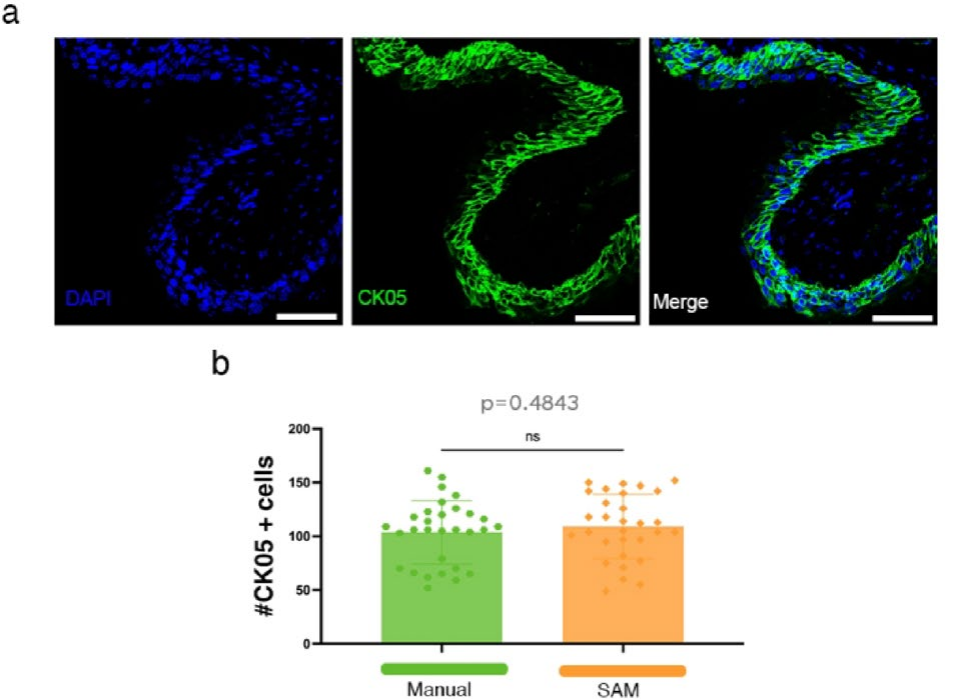
Comparison of CK05 + cell quantification methods in the bladder epithelium. (**a**) Representative immunofluorescence images of bladder epithelium stained for CK05 (green) and DAPI (blue). Scale bars: 50 µm. (**b**) Quantification of CK05 + cells using manual counting and SAM (n = 30 bladder sections).

To test if SAM can reliably perform quantifications beyond well-delimited structures, such as islets or SC-islets, and thus if it can be useful outside the islet biology field, we run it on murine bladder tissue. Urinary bladder is a reservoir that stores and empties urine in a coordinated and complex manner. There are four main layers of bladder, which includes urothelium, lamina propria, muscularis propria and serosa. The adult urothelium has three sub-populations that express different sets of markers^35^. 90% of the urothelial is cytokeratin-5 (CK-05) expressing cells residing in the basal layer. Intermediate and superficial (or umbrella) cells, on the other hand, each make up 5% of the urothelium and populate, respectively, in supra-basal and luminal layers^36^. Thus, murine bladder sections present a continuous multilayered configuration, without the formation of well-defined sub-structural structures characteristic of many other tissues or organs. We analyzed CK05 + cells (epithelial cells, Fig. 5a) in the bladder epithelium, where cells were counted within a single-layer structure.

As before, we compared manual counting with the SAM to determine the consistency between approaches. The quantification revealed no significant difference between the two methods (*p* = 0.4843), indicating that the automated analysis provides comparable results to traditional manual counting (Fig. 5b). These findings support the robustness of SAM method for epithelial cells in continuous fields.

### SAM can robustly cope with low quality samples

Low-quality samples, such as those with high background noise, can pose challenges for intensity-based approaches. To assess SAM’s performance under these conditions, we compared it to manual counting in low-quality samples of mouse islets of Langerhans.

**Fig. 6:**
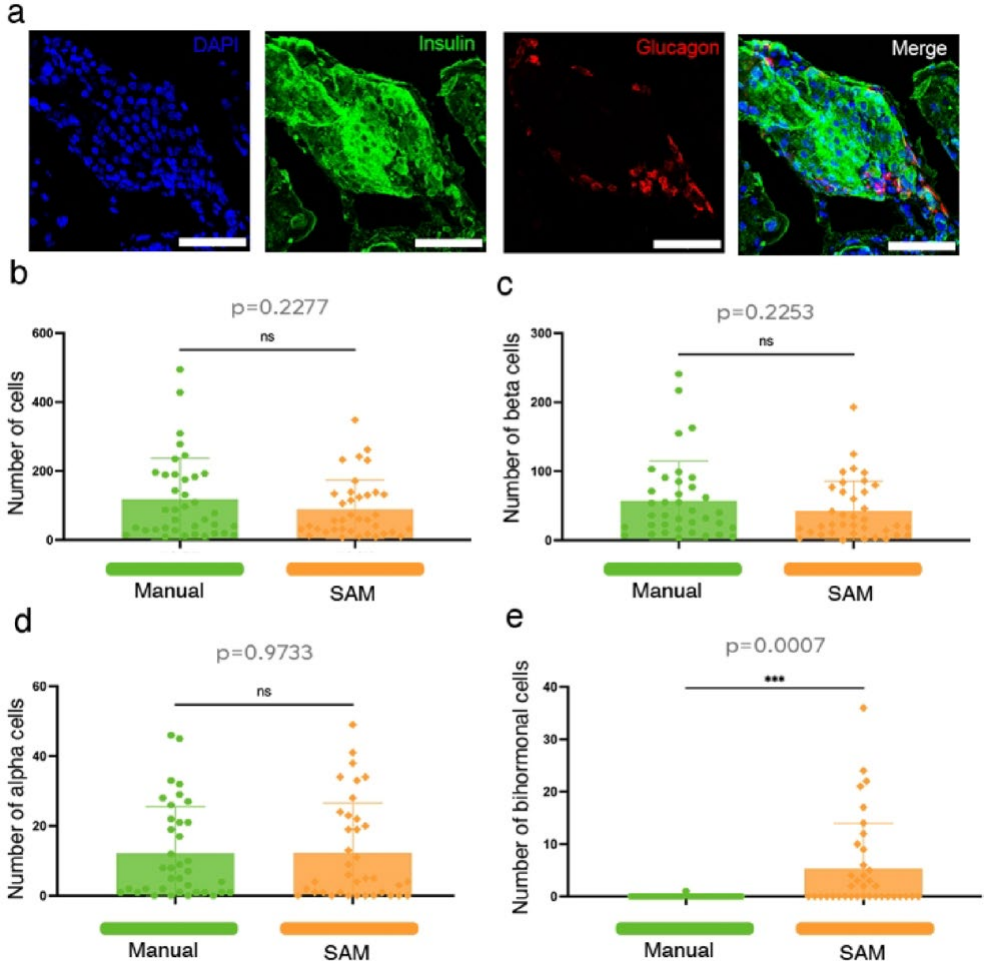
(**a**) Representative images of mouse islets of Langerhans, exhibiting high background staining. Nuclei are stained with DAPI (blue), insulin (green), and glucagon (red). Scale bars: 50 µm. (**b**) Quantification of the total number of cells per islet section, with cell counts obtained through both manual counting and SAM-based analysis (n = 37 islet sections). (**c**) Quantification of insulin-positive cells per islet section, comparing manual counting and SAM-based quantification (n = 37 islet sections). (**d**) Quantification of glucagon-positive cells per islet section, with both manual counting and SAM analysis methods (n = 37 islet sections). (**e**) Quantification of bihormonal cells (cells positive for both insulin and glucagon) per islet section, evaluated using manual counting and SAM-based quantification (n = 37 islet sections).

Despite high background levels (Fig. 6a), SAM produced similar quantification results to manual counting for total cell numbers (Fig. 6b, *p* = 0.2277). Likewise, no significant differences were observed between SAM and manual counting for the β-cell population (Fig. 6c, *p* = 0.2253) or α-cells (Fig. 6d, *p* = 0.9733). However, when analyzing bihormonal cells^37^, a significant discrepancy emerged between SAM and manual counting (Fig. 6e, *p* = 0.0007).

These results indicate that SAM can reliable quantity monohormonal α- and β-cell populations in low-quality samples, even with high background noise. However, its performance declines when analyzing bihormonal cells, likely due to the dense cellular environment and close proximity of overlapping signals. This limitation becomes more pronounced in high-background conditions, where distinguishing individual signals is increasingly difficult. While SAM remains a viable tool, manual validation is recommended in these cases.

### Increased efficiency of the SAM cell quantification

**Fig. 7:**
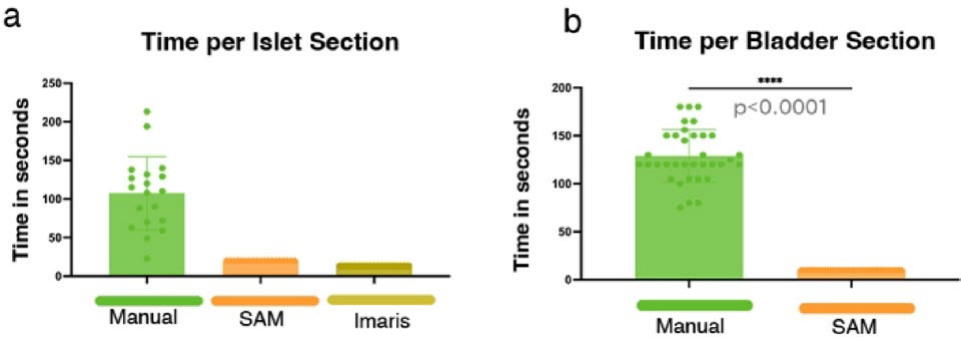
(**a**) Time required for manual counting, SAM, and Imaris-based quantification per islet section (n = 19 islet sections). (**b**) Time required for manual counting vs. the Macro method per bladder section (n = 37 bladder sections).

A key advantage of the SAM method is its substantial reduction in analysis time compared to manual counting. We quantified the time required per tissue section for both islets (Fig. 7a) and bladder sections (Fig. 7b). In both cases, SAM significantly outperformed manual counting, demonstrating a dramatic reduction in processing time. Specifically, in bladder sections, the Macro method was significantly faster than manual counting (*p* < 0.0001). These findings highlight the efficiency of SAM approach in large-scale tissue analysis.

### SAM exhibits clear limitations in the robust identification and quantification of rare events

To evaluate SAM reliability in rare events quantification, we assessed the presence of bihormonal glucagon + /insulin + cells in the pancreatic islets. In homeostatic conditions, the pancreatic neuroendocrine cells are monohormonal (i.e. secrete exclusively one hormone type), with little to none being bihormonal or polyhormonal. Following diverse types of stressors or during diabetes, the number of bihormonal cells increases significantly^33^, despite still being considered a rare event. One such example is the increment in the number of glucagon + /insulin + cells in the HNF1A mutant.

**Fig. 8:**
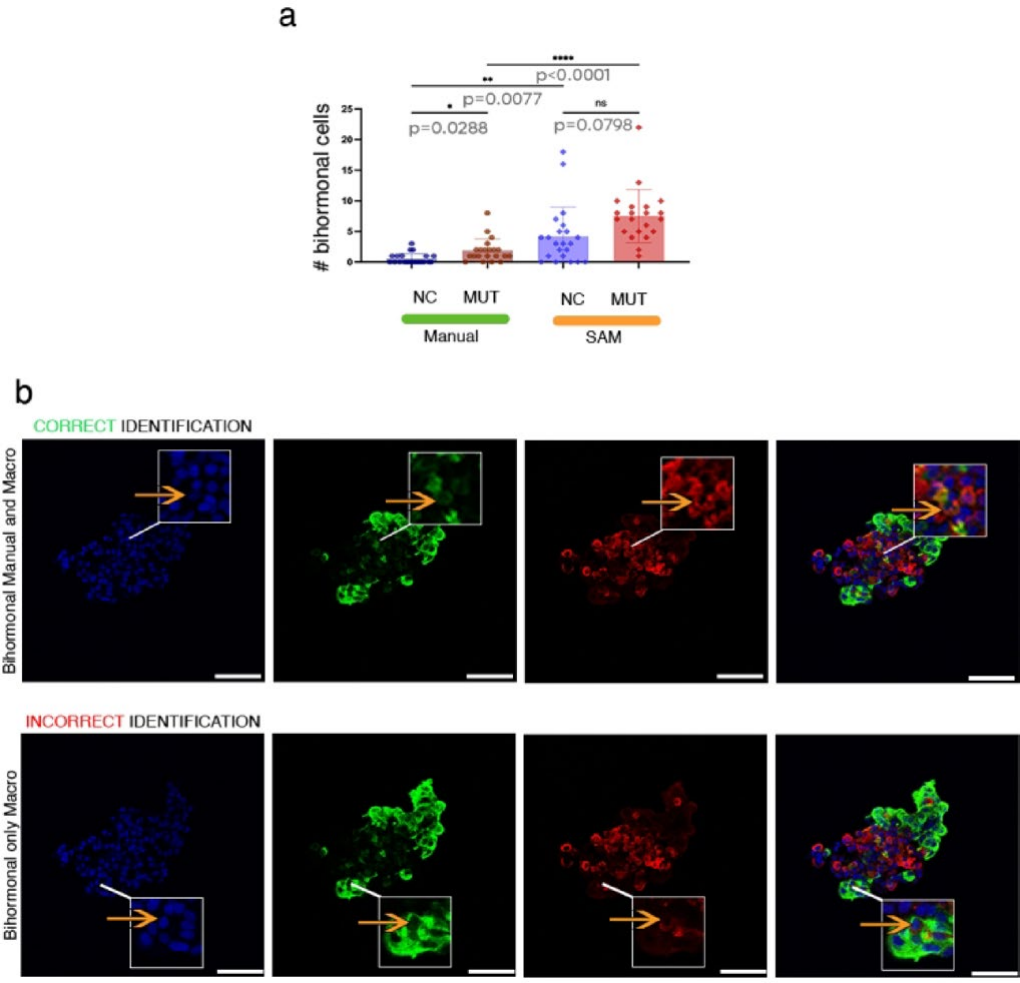
(**a**) Quantification of bihormonal cells in SC-islets from both control (NC) and mutant (MUT) conditions using two approaches: manual counting and automated analysis via the SAM macro (n = 22 sc-islet sections for NC and n = 21 sc-islet sections for MUT). (**b**) Representative Zooms of SC-islet in Fig. 2 to illustrating bihormonal cells. Cells correctly identified by both manual and SAM-based quantification, while cells detected only by the macro are considered false positives. Staining includes DAPI (blue) for nuclei, insulin (green), and glucagon (red). Scale bars: 50 µm.

The comparison between manual counting and SAM revealed a significant overestimation of the bihormonal cell numbers by SAM quantification (Fig. 8a) for both control (*p* = 0.0077) and mutant (*p* < 0.0001) images, while failing to detect the difference between the two conditions (*p* < 0.0288^[manual]^, 0.0798^**[SAM**]^). These errors were caused by the misidentification of overlapping objects in the two relevant confocal channels as bihormonal cells (Fig. 8b).

## Discussion

We present here a new user-friendly macro (SAM) using Fiji (ImageJ) to automatically quantify cytoplasmic expressing cells. Briefly, by comparing with manual counting (gold standard), open-source and commercial imaging software, we show that SAM quantification is robust for cell population quantification in most tested conditions. Our tests indicate that SAM is particularly adequate for pancreatic islet cell population quantifications, which due to its high cell density, usually presents challenges for automated tools. Moreover, the possibility of quantifying cells according to their signal intensity using user-imposed threshold is a plus, as many developmental processes involve the crosstalk between cell populations with low/high protein signal, instead of a simple absent/present staining situation. SAM is easy to install and employ by cell biologists with no coding skills, while also allowing a high level of customization and adaptation for more specific needs.

Quantifying populations with varying intensities is extremely challenging for human observers and often leads to unreliable results due to subjectivity and variability in interpretation. SAM offers a robust solution to this issue, enabling accurate and consistent quantification of different intensity populations in a reliable manner. However, as with all intensity measurements it is essential that the sample quality remains high, and that the same recording settings are maintained across comparisons.

SAM’s segmentation and classification strategy results in a systematic overestimation of double-positive cell counts, primarily due to the misinterpretation of overlapping signals as genuine co-expression. Addressing the limitation will require fine tuning of the threshold specifically for double- and multi-expressing cells. Specific threshold for multi expressing cell types, would reduce false positives and SAM’s performance could be brought closer to manual accuracy.

While our macro offers advantages in certain conditions, it is important to note that its performance is optimized for specific scenarios. Our method provides benefits over manual counting by reducing subjective bias, significantly decreasing analysis time, and ensuring consistent quantification criteria across samples. However, its advantage is context-dependent and may not universally outperform other techniques in all situations, especially when quantifying rare events in dense tissues.

## Methods

### SAM FIJI

Images must be in TIFF format for processing with SAM, a semi-automated FIJI-based macro^38^ designed to quantify cytoplasmic expression in multi-channel immunofluorescence images. The macro supports up to five channels and processes both single-plane and Z-stack images. For Z-stacks, users can choose between “Max Intensity” and “Mean Intensity” projections based on their analysis needs.

### ROI selection and segmentation

Before segmentation, users have the option to manually define a Region of Interest (ROI) to isolate specific areas for analysis. Nuclei are then segmented using Channel 1 (typically DAPI) with the StarDist2D plugin^13,39,40^, which provides precise nuclear segmentation.

### Quantification

For each selected channel, SAM generates two sets of measurements: one using the original ROI and another with a 1 µm enlargement to account for surrounding cytoplasmic expression. Both sets of measurements are saved as separate CSV files for each image and channel.

### Data processing (SAM_R_Script)

To process the data, a separate R script must be run. Before starting, users must configure the analysis by selecting which channels to include and setting threshold values. This configuration is saved as a YAML file, which must be created before running the script and can be reused for future analyses to maintain consistency. The script processes each pair of “without_enlargement” and “with_enlargement” files and calculates a new mean intensity for each cell.

Cells are classified as positive for a given channel if their new mean intensity exceeds the threshold defined in the configuration (Table 1). To establish thresholds, users manually measured the intensity they considered representative for the given cell type. This value was then multiplied by 0.6 to define the final threshold. For detailed instructions on how thresholds should be determined, refer to the SAM guidelines (Supplementary File 1).

**Table 1:** Thresholds for SAM quantification.

C-peptide (SC-islets)	40
Insulin (mouse-islets)	150
Glucagon (mouse-islets)	30
Glucagon (SC-islets)	50
High glucagon (SC-islets)	150
High C-peptide (SC-islets)	150
CK05	30

### Summary and output

The script compiles a summary for each sample, including total cell counts, the number of cells positive for each channel, cells positive for only specific channels, and those positive for combinations of channels. It also identifies cells that do not express any of the selected markers. The final summary, including counts and percentages, is saved as a CSV file in the specified output folder.

For more details read the SAM instructions (Supplementary File 1).

### Cell source and in vitro differentiation

The cell lines used in this study were provided by Synthego, as commercially available induced pluripotent stem cells (iPSC) generated by retroviral reprogramming of skin fibroblasts from the PGP1 donor from the Personal Genome Project (PGP) (Coriell, GM23338), or modified to introduce a cytosine ((CCA- > CCCA) in the polycytidine tract of codon 291 (p291fsinsC) of HNF1α, generating the HNF1AP291fsinC heterozygous knock in cell clones. hiPSCs were maintained on plates coated with Geltrex LDEV-Free Reduced Growth Factor (Gibco, A1413202) in mTeSR Plus cGMP stabilized feeder-free maintenance medium (Stem Cell Technologies, 100–0276) as described previously. For passaging, hiPSC colonies were detached after treatment with Gentle Cell Dissociation Reagent (StemCell Technologies, 100–0485). Prior to differentiation induction, the hiPSC cultures were confirmed to be mycoplasma-free using the MycoAlert Mycoplasma Detection Kit (Lonza, LT07-418). The hiPSCs were differentiated in triplicates according to a previously published stepwise protocol^16,41^ by seeding 1,500,000 cells/well in Geltrex-coated 6-well plates.

### SC-islet preparation for imaging

Harvested SC-islets were fixed in 4% PFA in PBS overnight at 4 °C, followed by dehydration in a sucrose gradient, up to 30%. The sucrose was then replaced with Tissue Tek OCT (Sakura) and kept overnight at 4 °C. The SC-islets were moved to embedding molds, covered with Tissue Tek O.C.T, and flash-frozen in beakers surrounded by liquid nitrogen. Frozen tissue blocks were sectioned at 5-µm on Superfrost Plus Microscope Slides (Epredia) using a cryostat (Leica CM1950, Germany).

### Murine models

The following mouse strains were used B6(129)-Tg(Upk2-cre)1Rkl/WghJ (Jax strain number 029281), B6.129X1-Gt(ROSA)26Sortm1(EYFP)Cos/J (Jax strain number 006148) and Tg(Gcg-cre)1Herr on a mixed C57BL6J/CBA genetic background, the latter being kindly donated by Prof. Pedro Herrera (University of Geneva, Switzerland).

Animals were housed in a pathogen-free animal facility, in groups of 2 to 5 in internally ventilated cages (IVC II) under controlled conditions (22 °C, 12-h light/dark cycle) with ad libitum access to water and standard diet RM1A (SDS). All breeding and experiments were approved by the Norwegian Animal Research Authority under ethical approval licenses: FOTS 29,087, 29,088, 25,526 and 25,531. Animal care and handling adhered to the guidelines set by the Norwegian Animal Research Authority and complied with the European Union Directive 2010/63/EU. The study is reported in accordance with ARRIVE guidelines.

Mice were sacrificed by cervical dislocation without anesthesia by trained experienced personnel, death confirmed by separation of the spinal cord from the skull. Bladders and pancreases were collected from 12 weeks old male and female mice as previously described^42,43^. Altogether 10 animals were used for the study (6 animals for streptozotocin studies; 4 animals for bladder studies).

### Streptozotocin administration

The streptozotocin (STZ) dosage was determined based on a previously described protocol^32^. Female mice were fasted for 4 h prior to STZ or vehicle administration (3 mice per condition). A single-dose injection of STZ (Sigma, S0130) was prepared in 0.1 M citrate buffer (pH 4.5) to achieve an injection dosage of 100 mg/kg. Control animals received an equivalent volume of the citrate buffer vehicle.

### Immunofluorescence staining

Cryostat sections (10 μm) of bladder, stem cell islets and pancreas were stained with primary antibodies against cytokeratin-5 (1:400, Antibodies Online, ABIN3184234), c-peptide (1:200, DSHB, GN-ID4), glucagon (1:1000, Sigma Aldrich, G2654) and insulin (1:400, Geneva Antibody Facility, ABCD_AE804) respectively. Secondary antibodies were anti-rabbit IgG A488 (1:500, Thermo Fisher, A21206), anti-mouse IgG1a A546 (1:500, Molecular Probes, A-11074) or donkey anti-guinea pig IgG 488 (1:500, Thermo Fisher, A-11073), with DAPI (Invitrogen, P36931) as a nuclear counterstain. Sections were mounted in ProLong Diamond (Invitrogen, P36931) and imaged using a Leica SP8-STED confocal microscope.

### Imaris volumetric analysis

Acquisition of insulin volume for the STZ protocol was done using volume algorithm in the Imaris 9.2.1 program (Andor technology)^44^. Parameters used for automatic volumetric counting can be found in Table 2.

**Table 2:** STZ insulin volume Imaris algorithm parameters.

Enable region of interest	False
Enable region growing	False
Enable tracking	False
Source channel index	2
Enable smooth	True
Surface grain size	1.00 um
Enable eliminate background	False
Diameter of largest sphere	2.26 um
Enable automatic threshold	True
Active threshold	True
Enable automatic threshold B	True
Active threshold B	True
“Number of voxels Img = 1”	Above 5000

### QuPath quantification

QuPath Version 0.4.3: Open-source software platform^25^ was used to count the number of Dapi + , c-peptide + and glucagon + cells on stained sections of stem cell derived islet like organs. First, Image type was set to Fluorescence upon opening image and Channels were separated by name using Brightness/contrast command. Islet was identified by manually drawing around it using annotations tool to detect the area of interest for each image. Analyze → cell detection → Positive cell detection command was used with the following settings: detection channel, Dapi; Pixel size,0.5 μm; Nucleus parameters, Background radius, 8 μm; median filter radius, 0 µm; sigma, 1.5 µm; minimum cell area, 10 µm^2^; maximum cell area, 400 µm^2^; Intensity parameters, threshold, 25; Cell parameter, cell expansion, 5 μm With Threshold 1 + ,2 + , and 3 + set to 10,20 and 30 to detect varying intensities.

### Statistical analyses

Statistical analyses were performed using GraphPad Prism v10.4.1 (GraphPad Software Inc., USA). To assess the statistical differences between groups for the immunofluorescence data quantification and physiological parameters we used t-test, unless otherwise specified in the corresponding figure legend. In figures, data are represented as mean ± SEM (standard error) unless otherwise specified. Statistical significance was defined at *p* < 0.05 ( ∗), *p* < 0.01 (∗ ∗), *p* < 0.001 (∗ ∗ ∗), and *p* < 0.0001 (∗ ∗  ∗ ∗).

## Electronic supplementary material

Below is the link to the electronic supplementary material.

Supplementary Material 1

## Data Availability

The source code of SAM along with all documentation has been deposited in GitHub and is freely accessible at https://github.com/LucasUnger/SAM.
