# Supplementary material for: Efficient cytoplasmic cell quantification using a semi-automated FIJI-based tool
Source: Sci Rep. 2025 Jul 28;15:27509. doi: 10.1038/s41598-025-12144-x (PMC12304148; doi:10.1038/s41598-025-12144-x)
Supplement: Supplementary file 1 — Supplementary Material 1 [file 41598_2025_12144_MOESM1_ESM.pdf]

Efficient Cytoplasmic Cell Quantification using a Semi-Automated FIJI-Based Tool

Lucas Unger[1,*], Ulrik Larsen[1], Shayla Sharmine[1], Md Kaykobad Hossain[2], Thomas Aga Legøy[1], Marc Vaudel [1,3], Luiza Ghila[1], and Simona Chera[1,*]

Affiliations:

[1] Mohn Research Center for Diabetes Precision Medicine, Department of Clinical Science, Faculty of Medicine, University of Bergen, Glasblokkene 1, Haukelandsbakken 15, 5009 Bergen, Norway

[2] Department of Clinical Science, Faculty of Medicine, University of Bergen, Postboks 7804, 5020 Bergen, Norway

[3] Computational Biology Unit, Department of Informatics, University of Bergen, Bergen, Norway

*To whom correspondence should be addressed:
simona.chera@uib.no
lucas.unger@uib.no

Supplemental information: SAM Instructions

Prerequisites:

- Installed **FIJI** (ImageJ 2)
- R-Language (R-Studio) installed
- Images to analyse need to be in 8-bit and Tiff format
- You **NEED** to have a nuclear staining (e.g: DAPI) in the First channel of your images
- Install StarDist and CSBDeep in FIJI (https://imagej.net/plugins/stardist) (more info)

Before running the Macro:

- Make sure you have good Images to analyze (little-no oversaturation, little-no Background)
- All Tiff you want to analyze should be in one Folder and all CSV created by the FIJI Macro should be saved in a separate Folder
- Determine the threshold for your channels (see How to determine threshold)
- Check what the best Stardist segmentation settings are for your nuclear Staining (more Info in Stardist segmentation)

Running the Analysis Pipeline:

Start by running your image analysis in Fiji:

1. Simply drag and drop "TheMacroToRuleThemAll.ijm" into the Fiji window. While the macro runs, check if the segmentation results and DAPI nuclei ROIs look correct - if they appear unusual, there might be issues with your image preparation or file format
2. In the first dialog window, select your input folder containing all 8-bit TIFF files
3. In the second dialog window, choose where you want to save the CSV output files

Processing with R

After the Fiji analysis is complete:

1. Launch "RscriptforTheMacroToRuleThemAll.R"
2. Select the folder containing your CSV files when prompted
3. Choose the output location for the configuration and summary files
4. Enter your threshold values in the designated field
5. **Important**: Save your configuration before proceeding - the script won't run without a saved configuration
6. Click Run to start the analysis

Install StarDist and CSBDeep:

Installation

1. Start Fiji (or download and install it from here first).
2. Select `Help > Update...` from the menu bar.
3. Click on the button `Manage update sites`.
4. Scroll down the list and tick the checkboxes for update sites `CSBDeep` and `StarDist`, then click the `Close` button. (If `StarDist` is missing, click `Update URLs` to refresh the list of update sites.)

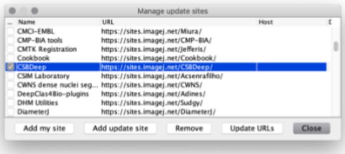

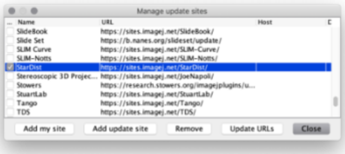

5. Click on `Apply changes` to install the plugin.
6. Restart Fiji.

<u>How to determine threshold:</u>

Setting Up Your Analysis

Setting appropriate thresholds is a key step in achieving reliable image analysis results. While you can determine these values manually by examining representative images from your experiment, automated approaches like Otsu's method can also help establish objective cutoff values.

How This Macro Works

This analysis requires threshold determination for each channel, except for the nuclear staining in Channel 1. The macro evaluates the average signal intensity within a 1 µm region around each nucleus - if this intensity exceeds your set threshold, the cell is classified as positive.

Important Considerations

Since ROIs don't always perfectly align with cellular boundaries, consider adjusting your threshold calculations accordingly. A good practice is to set your positive signal threshold at 50-60% of typical cellular expression levels (i.e., multiply your standard threshold by 0.5-0.6). This adjustment helps account for cases where the ROI only partially overlaps with a cell while still maintaining reliable detection of positive signals.

Your threshold settings should align with your experimental goals - whether focusing on highly expressing populations or capturing a broader range of signal intensities.

Best Practices

- Test your thresholds on multiple representative images
- Maintain consistent settings throughout your analysis
- Document your chosen threshold values

- Adjust criteria based on your specific experimental requirements

Remember that the quality of your results depends on appropriate threshold selection and consistent application of these criteria throughout your analysis.

Example

To determine the appropriate threshold for quantifying cytoplasmic cells, I measured the background intensity in 10 different areas and obtained an average value of 32, with the highest being 41. I then calculated the background threshold by multiplying the average (32) by 1.5, resulting in a value of 48. Since the maximum measured background (41) does not exceed this threshold, I am using 48 as the treshhold in my R script for cell quantification.

<u>Stardist segmentation</u>

Nuclear Segmentation with StarDist

StarDist's segmentation quality is crucial as it forms the foundation for all subsequent analysis by defining your cell boundaries. The method uses star-convex shapes to detect and separate nuclei, which works particularly well for densely packed and blob-like objects.

Checking Your Segmentation

While the default settings in the macro should work well for most nuclear staining patterns, it's essential to verify the segmentation quality with your specific samples. Pay attention to:

- Whether nuclei are properly separated
- If the nuclear boundaries are accurately detected
- Whether the algorithm misses dim nuclei or creates false positives

Optimizing Detection

If you notice segmentation issues, consider adjusting these key parameters:

- Probability threshold: affects how confident the model needs to be to detect a nucleus
- NMS threshold: controls overlap between detected objects
- Input image normalization helps with varying staining intensities

For optimal results, ensure your nuclear staining (DAPI/Hoechst) has sufficient signal intensity and good contrast. Poor staining quality or very low exposure times can lead to suboptimal segmentation results.

Remember that accurate nuclear segmentation is critical since all subsequent measurements and analysis depend on proper cell identification.

To help with setting up StarDist parameters for your specific needs, you can use Fiji's macro recorder (Plugins > Macros > Record) while running StarDist on just your nuclear channel (make sure to close all other channels first). The recorded command can either be modified at line 79 in the existing macro or replaced with your new settings using the "Command From Macro" syntax when running StarDist.

```
75      // Process Channel 1 (DAPI) for segmentation
76      selectWindow(projectionWindow);
77      run("Duplicate...", "title=Channel_1 duplicate channels=1");
78      rename("DAPI");  // Ensure Channel 1 is renamed to DAPI
79      run("Command From Macro",
80          "command=[de.csbdresden.stardist.StarDist2D], " +
81          "args=['input':'DAPI', 'modelChoice':'Versatile (fluorescent nuclei)', " +
82          "'normalizeInput':'true', 'percentileBottom':'1.0', 'percentileTop':'99.8', " +
83          "'probThresh':'0.7500000000000001', 'nmsThresh':'0.4', " +
84          "'outputType':'ROI Manager', 'nTiles':'1', 'excludeBoundary':'2', " +
85          "'roiPosition':'Automatic', 'verbose':'false', 'showCsbdeepProgress':'false', " +
86          "'showProbAndDist':'false'], process=[false]");
87
```

<u>Know problems:</u>

MacOS users experience problems with StarDist segmentation.
To fix this open FIJI

Edit › Options › TensorFlow

And change the version to TF 1.12.0 CPU, afterwards restart and it should work.
